# Transcranial magnetic stimulation facilitates neurorehabilitation after pediatric traumatic brain injury

**DOI:** 10.1038/srep14769

**Published:** 2015-10-06

**Authors:** Hongyang Lu, Tali Kobilo, Courtney Robertson, Shanbao Tong, Pablo Celnik, Galit Pelled

**Affiliations:** 1 F. M. Kirby Research Center for Functional Brain Imaging, Kennedy Krieger Institute, Baltimore, MD, USA; 2 The Russell H. Morgan Department of Radiology and Radiological Science, The Johns Hopkins University School of Medicine, Baltimore, MD, USA; 3 School of Biomedical Engineering, Shanghai Jiao Tong University, Shanghai, China; 4 Department of Anesthesiology/Critical Care Medicine and Pediatrics, The Johns Hopkins University School of Medicine, Baltimore, MD, USA; 5 Department of Physical Medicine and Rehabilitation, The Johns Hopkins University School of Medicine, Baltimore, MD, USA

## Abstract

Traumatic brain injury (TBI) is the leading cause of death and disability among children in the United States. Affected children will often suffer from emotional, cognitive and neurological impairments throughout life. In the controlled cortical impact (CCI) animal model of pediatric TBI (postnatal day 16–17) it was demonstrated that injury results in abnormal neuronal hypoactivity in the non-injured primary somatosensory cortex (S1). It materializes that reshaping the abnormal post-injury neuronal activity may provide a suitable strategy to augment rehabilitation. We tested whether high-frequency, non-invasive transcranial magnetic stimulation (TMS) delivered twice a week over a four-week period can rescue the neuronal activity and improve the long-term functional neurophysiological and behavioral outcome in the pediatric CCI model. The results show that TBI rats subjected to TMS therapy showed significant increases in the evoked-fMRI cortical responses (189%), evoked synaptic activity (46%), evoked neuronal firing (200%) and increases expression of cellular markers of neuroplasticity in the non-injured S1 compared to TBI rats that did not receive therapy. Notably, these rats showed less hyperactivity in behavioral tests. These results implicate TMS as a promising approach for reversing the adverse neuronal mechanisms activated post-TBI. Importantly, this intervention could readily be translated to human studies.

Traumatic brain injury (TBI) due to sports injuries, playground activities, vehicle injuries, falls and assault is the leading concern in children and young adults in the United States. Almost half a million emergency department visits for TBI are made annually by children aged 0 to 14 (Centers for Disease Control and Prevention). TBI results in immediate injury from direct mechanical forces. Secondary injury results from altered cerebral blood flow and the release of biochemical and inflammatory factors which can interfere with normal vascular, anatomical, neuronal and glial physiology in the acute, subacute and chronic phases^1^. Current therapies are focused on minimizing acute post-injury excitotoxicity, cerebral edema, mitochondrial injury and neuronal inflammation by various methods, including controlled hypothermia and hypertonic saline^2^. Chronic rehabilitative care is focused on maintaining and advancing medical stability, treating related co-morbid conditions such as pain management, and tone and movement disorders, sleep disorders, hydrocephalus and psychiatric disturbances; as well as relearning skills that were lost or compromised^3^,^4^.

Nevertheless, up to 48% of affected children suffer from emotional, cognitive and neurological impairments that persist well into adulthood^5^,^6^,^7^,^8^. Accumulating evidence suggests that injury to the developing brain induces both long-term anatomical and functional changes in the neuronal network and at the cellular level: Neuronal loss and white matter disruptions throughout the brain are often observed in human and animal models of TBI^9^,^10^,^11^; Abnormal neuronal plasticity that is manifested in increased seizure vulnerability^12^,^13^,^14^, inappropriate neuronal rewiring^15^, and atypical neuronal responses^11^,^16^ are common post-injury occurrences. We have recently demonstrated in a rodent model of pediatric TBI, that injury results in long-term impaired plasticity in remote, non-injured cortical areas^11^. This was exhibited by a significant decrease in the ability to induce long-term potentiation (LTP) which is one of the major cellular mechanisms of plasticity, as well as in neuronal hypoactivity. The latter was manifested by decreases in evoked neuronal responses that were measured with multi-unit activity (MUA), local field potential (LFP), and functional magnetic resonance imaging (fMRI) in neurons located in the contralateral, non-injured cortex. Acute and chronic neuronal hypoactivity following TBI was previously implicated as a contributing factor to post-traumatic epileptogenesis in rodent models^17^,^18^,^19^ and may delay and prohibit normal circuit maturation and recovery during development^20^, thus contributing to the neurological and cognitive deficits that TBI victims suffer.

Capitalizing on these studies, reshaping the abnormal post-injury neuronal activity materializes as a suitable strategy to augment rehabilitation. Non-invasive brain stimulation technologies such as transcranial magnetic stimulation (TMS), can induce changes in neural excitability that outlast the period of stimulation via induction of weak electric currents using a rapidly changing magnetic field. TMS has shown preliminary success as a therapeutic tool in the clinic in adult neurological diseases such as stroke, epilepsy, major depression and migraines^21^,^22^ and provides relief from the chronic pain associated with a variety of injuries^23^,^24^,^25^. In addition, TMS has been proposed as a possible treatment for adult TBI^26^,^27^,^28^. However, the potential to use TMS as a rehabilitative strategy in children and young adults who suffer from TBI has yet to be explored.

Here we investigated whether rescuing neuronal activity by TMS therapy would alleviate long-term neuronal and behavioral impairments in the controlled cortical impact (CCI) rat model of pediatric TBI. The CCI model recapitulates clinically relevant histopathological, neurophysiological and behavioral characteristics of moderate-to-severe-pediatric TBI and is used to study treatment strategies in head injuries^29^,^30^,^31^,^32^,^33^. TMS was delivered at a high frequency of 20 Hz, a paradigm known to induce focal excitatory neuronal changes in humans^34^. TMS therapy began in the subacute stage and was delivered twice a week over a 4-week period, as previous studies demonstrated that starting aggressive rehabilitation early had a positive impact on the functional status of the patient^35^. Biomarkers for neuronal activity in the form of expression of plasticity markers and electrophysiological and fMRI responses as well as behavioral testing were evaluated in the weeks after the therapy ended to assess the long-term functional outcome.

## Results

### TBI and TMS

TBI over the left somatosensory cortex was induced using a CCI device on 20 Sprague-Dawley rats at postnatal day 16–17 (30–45 g). This age range is believed to be equivalent to the toddler age in humans^30^,^36^. An additional six rats did not undergo any surgery and served as age-matched controls. The TMS coil was placed over the non-injured S1 using a custom-built holder. TMS consisted of nine trains of 100 pulses delivered at a frequency of 20 Hz and an inter-train rest time of 55 s to allow effective cooling of the coil. This protocol was applied in TBI rats (*n* = *10*; TBI + TMS group) twice a week, starting nine days after the TBI procedure, for four weeks. The control groups consisted of TBI rats (*n* = *10*; TBI group) and healthy rats (*n* = *6*; control group) that were anesthetized and secured in a stereotaxic frame for the same length of time as the experimental group, and although the TMS coil was positioned over the right hemisphere, TMS pulses were not delivered. In order to determine the long-term effect of TMS therapy on pediatric TBI subjects, the neurophysiological and behavioral assessments were performed three days (fMRI), four days (behavior), and 7–14 days (electrophysiology) after the last TMS therapy session. At the end of the electrophysiology recordings, animals were sacrificed for further immunostaining processing.

### TMS promotes CaMKII expression after TBI

**Figure 1:**
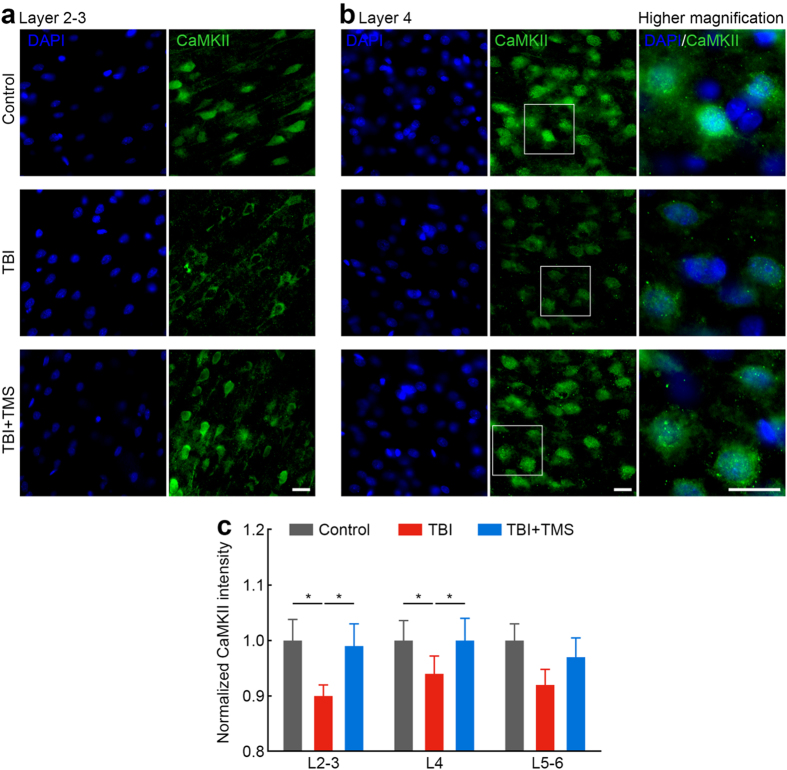
TMS induces synaptic plasticity after TBI. Immunofluorescence staining with nuclear (DAPI) and synaptic plasticity (CaMKII) markers show that TBI rats that received four-week TMS treatment had an increased expression of CaMKII as is indicated by increased fluorescent intensity, compared to TBI rats not subjected to the treatment. Increases in CaMKII fluorescent intensity were found in L2–3 (**a**) and L4 (**b**). Higher magnification images of the insets are shown in the right panels. Scale bars: 20 μm. (**c**) Group data shows increases in CaMKII intensity in TBI + TMS rats (*n* = *2*), compared to TBI rats (*n* = *2*; *P < 0.05). For each rat, three brain slices around bregma 0 were selected for staining. Data was calculated from six brain slices for each group with a field of view of 0.04 mm^2^.

To determine whether TMS induced synaptic plasticity, we calculated the expression levels of Ca^2+^/calmodulin-dependent kinase II (CaMKII), a gene known to be involved in LTP. The fluorescence intensity of CaMKII was calculated and normalized to data from control rats. Figure 1a,b shows representative immunofluorescence staining in layers 2–3 (L2–3) and layer 4 (L4) of non-injured S1, respectively. Figure 1c shows the normalized CaMKII intensity across the non-injured S1 cortical layers. Brain slices were also stained for nuclear marker (DAPI). Calculation of the number of cells demonstrated that the TBI injury did not induce any cell loss in the non-injured S1 (Table 1). However, there were statistically significant differences in the fluorescence intensity of CaMKII between groups as determined by one-way analysis of variance (ANOVA, L2–3 *F*(2,15) = 3.850, *P* = 0.045; L4 *F*(2,15) = 3.705, *P* = 0.049; Layer 5 (L5) *F*(2,15) = 3.495, *P* = 0.057). Post-hoc testing revealed that TBI decreased the CaMKII intensity in the non-injured S1: L2–3 10.0%, *P* < 0.05; and L4 6.0%, *P* < 0.05, compared with control rats. After four-week TMS therapy, significant increases in CaMKII intensity were found in the non-injured S1: L2–3 10.0%, *P* < 0.05; and L4 6.4%, *P* < 0.05, compared to TBI rats, suggesting that the TMS treatment induced post-injury synaptic plasticity.

### TMS restores cortical excitability after TBI

**Figure 2:**
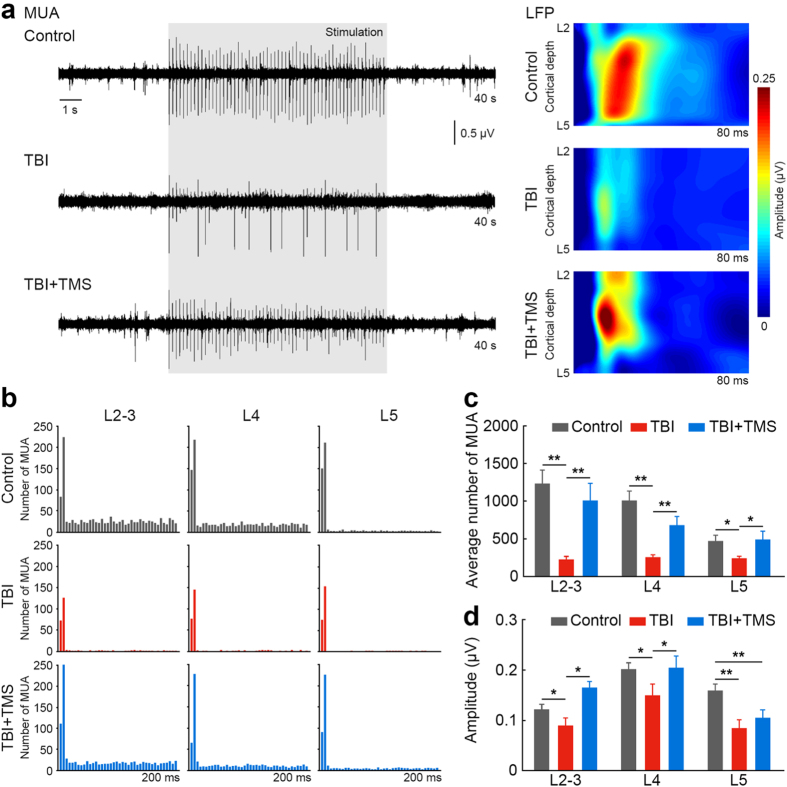
TMS restores cortical excitability after TBI. Increase in stimulus-evoked electrophysiology responses in the intact, non-injured S1 after four-week TMS treatment in TBI rats. (**a**) The representative MUA recordings show enhanced responses in L4 of non-injured S1. Gray box represent duration of tactile stimulus. LFP maps of representative control, TBI and TBI + TMS rats show an increase in the amplitude of stimulus-evoked LFP response across the S1 depth in a TBI + TMS rat, compared to a TBI rat. (**b**) Post-stimulus time histograms of representative control, TBI and TBI + TMS rats show an increase in the number of MUA responses across the S1 depth in a TBI + TMS rat, compared to a TBI rat not subjected to TMS therapy. (**c**) The average number of MUA responses across the S1 depth shows an increase in L2–3, L4 and L5 in TBI + TMS rats (*n* = *10*), compared to TBI rats (*n* = *10*; *P < 0.05; **P < 0.01). (**d**) The averaged amplitude of stimulus-evoked LFP response across the S1 depth shows an increase in L2–3 and L4 in TBI + TMS rats (*n* = *10*), compared to TBI rats (*n* = *10*; *P < 0.05; **P < 0.01). Recording was performed once for each individual animal.

Electrophysiology signals were recorded in response to contralateral tactile limb stimulation throughout the cortex using a 12-channel axial array microelectrode (FHC, Inc., Maine, USA). MUA responses reflecting the averaged spiking activity of neurons in the vicinity of the electrode were measured across the depth of the non-injured S1. One-way ANOVA showed statistically significant differences between groups (L2–3 *F*(2, 23) = 9.652, *P* < 0.001; L4 *F*(2,23) = 15.403, *P* < 0.001; L5 *F*(2,23) = 3.633, *P* = 0.043). Post-hoc testing revealed that TBI resulted in significant decreases of MUA responses across the layers of the non-injured S1: L2–3 229 ± 40, *P* < 0.01; L4 256 ± 34, *P* < 0.01; and L5 241 ± 28, *P* < 0.05 (*n* = *10*), compared to controls: L2–3 1231 ± 182, L4 1011 ± 125 and L5 469 ± 80 (*n* = *6*). Four-week TMS therapy significantly increased in the number of MUA responses across S1 layers: L2–3 1013 ± 225, *P* < 0.01; L4 679 ± 120, *P* < 0.01; L5 490 ± 110, *P* < 0.05 (*n* = *10*), compared to TBI rats that received no therapy. On average, TMS therapy increased the number of MUA responses in the TBI + TMS group compared to the TBI group by 200.6%. Figure 2a shows the representative MUA recordings in L4 of non-injured S1 in response to contralateral tactile limb stimulation. Figure 2b illustrates corresponding examples of post-stimulus time histograms of MUA responses in the different non-injured S1 cortical layers and Fig. 2c shows the MUA group averages.

In addition to the spiking activity, LFP reflecting the averaged synaptic activity was also measured. Statistically significant differences between groups were determined by one-way ANOVA (L2–3 *F*(2,23) = 9.072, *P* = 0.001; L4 *F*(2,23) = 3.449, *P*=0.049; L5 *F*(2,23) = 4.912, *P* = 0.017). TBI rats showed a decrease in LFP magnitude across the non-injured S1 cortical layers: L2–3 0.18 ± 0.03 μV, *P* < 0.05; L4 0.30 ± 0.04 μV, *P* < 0.05; and L5 0.17 ± 0.03 μV, *P* < 0.01, compared to control rats: L2–3 0.24 ± 0.02 μV; L4 0.40 ± 0.03 μV; and L5 0.32 ± 0.03 μV. After four-week TMS therapy, significant increases in LFP magnitude were found in the non-injured S1 layers of L2–3 0.33 ± 0.03 μV, *P* < 0.05; and in L4 0.41 ± 0.05 μV, *P* < 0.05; but not in L5 0.21 ± 0.03 μV, compared to TBI rats who did not receive the therapy. Figure 2a displays representative LFP magnitude maps from the different cortical layers in the non-injured S1 and Fig. 2d shows the LFP group averages.

### TMS improves evoked-fMRI cortical responses after TBI

**Figure 3:**
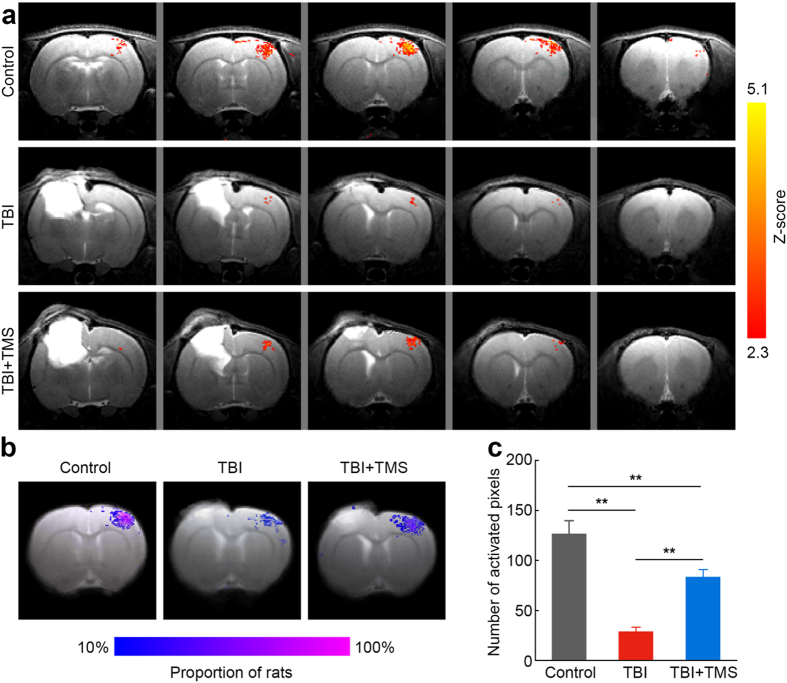
TMS improves evoked-fMRI cortical responses after TBI. Increase in BOLD fMRI responses in the non-injured S1 after four-week TMS treatment in TBI rats. (**a**) Representative BOLD fMRI Z-maps (*P* < 0.05) obtained from control, TBI, and TBI + TMS rats were overlaid on the T2-weighted, high-resolution anatomical images. The cortical damage resulting from TBI can be clearly visualized in the left hemisphere. Significant increases in fMRI responses to contralateral limb stimulation in the intact, non-injured S1 are observed after four-week TMS treatment, compared to controls and TBI rats not subjected to TMS therapy. (**b**) Incident maps of BOLD fMRI responses overlaid on averaged T2-weighted high-resolution anatomical images at bregma 0. Numbers of rats were normalized for control (*n* = *6*), TBI (*n* = *10*) and TBI + TMS (*n* = *10*) rats. (**c**) The average number of activated pixels across all the slices representing S1, shows an increase in evoked fMRI responses in TBI + TMS rats (*n* = *10*), compared to rats not subjected to TMS therapy (*n* = *10*; **P < 0.01). Imaging was performed once on each individual animal.

Functional neuroimaging techniques such as fMRI provide powerful tools for the non-invasive, *in vivo* examination of brain function. Indeed, the role of fMRI in assessing functional activation pattern after TBI in adults^37^,^38^,^39^ and children^40^,^41^ as well as in animal models^11^,^42^,^43^ has grown significantly over the past decade. Thus, fMRI offers a translatable and useful mean with which to test whether TMS therapy can restore post-injury neuronal activity. The CCI caused a significant architectural damage to the left somatosensory cortex, as can be observed in the MRI images (Fig. 3a). The damaged area did not show any neurophysiological responses to contralateral limb stimulation at any time point following the injury in the TBI and the TBI + TMS groups. For the non-injured S1, one-way ANOVA showed statistically significant in the number of activate pixels differences between groups as well (*F*(2,23) = 35.116, *P* < 0.001). In line with our previous observations^11^, TBI resulted in a significant decrease of 77.2% of fMRI responses not only in the injured S1, but also in the non-injured S1 compared to control rats (28.9 ± 4.6 in the TBI group, *n* = *10*, vs. 126.8 ± 13.0 in the control group, *n* = *6*; *P* < 0.01). In agreement with the electrophysiology results, four-weeks of TMS therapy significantly increased the number of activate pixels to 83.7 ± 7.2 in the non-injured S1 (*n* = *10; P* < 0.01), which was a 189.6% functional improvement compared to TBI rats that did not receive the TMS therapy. Figure 3a shows the fMRI activation Z-maps (*P* < 0.05) of representative rats overlaid on corresponding high-resolution T2-weighted MRI anatomical images (positioned at bregma −2–2 mm). Figure 3b depicts the incidents of fMRI responses in the center of S1 (bregma 0) across the entire group of rats in this study. This analysis shows the spatial distribution of the activated pixels in each of the groups, demonstrating a sparse distribution of the activated pixels in the TBI group, and a dense distribution of the activated pixels across all the individuals in the TBI + TMS group, as well as in the control group.

### Behavioral tests to assess hyperactivity after TBI

**Figure 4:**
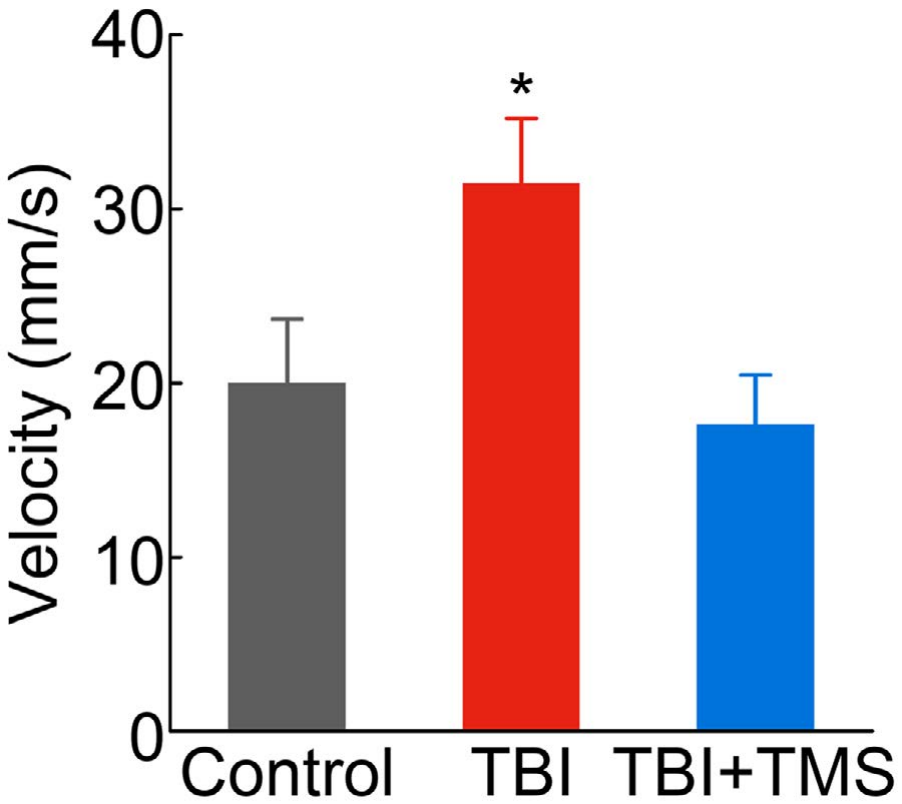
TMS reduces hyperactivity after TBI. TBI rats that received four-week TMS therapy exhibited less hyperactivity in the open-field test. The averaged velocity shows decreases in TBI + TMS rats (*n* = *10*), compared to TBI rats (*n* = *10*; *P < 0.05).

Results of the neurophysiology measurements offered a clear indication that TMS therapy could improve neuronal function in the non-injured S1. Nevertheless, one of the most important outcomes of a new therapy is its explicit effect on behavior. For this purpose rats were subjected to several behavioral tests to assess physiology and hyperactivity, the latter of which is a known complication in children who suffer TBI^44^,^45^. Rats’ forelimb and hindlimb reflexes were tested, but all groups showed normal reflexes to limb stretching (*n* = *5* in each group), suggesting that certain aspects of motor function recovered spontaneously after TBI. Hyperactivity is associated with pediatric animal models of TBI^46^ and has been tested using an open field apparatus. Animals were placed in the center of the arena and were allowed to move freely for 10 minutes while they were being video-recorded with an overhead camera. Consistent with previous reports^30^,^47^, the average velocity of TBI rats was 31.5 ± 3.7 mm/s (*n* = *10*) which was significantly higher compared to controls at 20.0 ± 3.7 mm/s; *n* = *6, P *< 0.05). In TBI rats that received the TMS therapy, the average velocity significantly decreased to 17.6 ± 2.9 mm/s (*n* = *10, P* < 0.05), compared to TBI rats not subjected to TMS therapy and was comparable to that of control rats (Fig. 4). The decrease in the velocity, which is indicative of hyperactivity, suggests that TMS therapy improved the hyperactivity disorder that has often been observed in patients^48^,^49^,^50^ and in animal models of pediatric TBI.

## Discussion

TMS is a popular tool for human brain stimulation and its application as a therapeutic strategy for neurological diseases such stroke, epilepsy, major depression and migraines^21^,^22^ has grown dramatically over the last several years. Based on the success of TMS and other non-invasive brain stimulation techniques to modulate brain activity in health and disease, TMS has been proposed as a possible treatment for adult TBI^26^,^27^,^28^. A recent study has shown that adult patients who suffered from mild TBI with various postconcussive symptoms (PCS), and who underwent TMS therapy, reported decreases in PCS scores, and demonstrated increases in task-related fMRI activation^51^. To date, TMS has been reportedly used in over 800 normal children and over 300 neurologically abnormal children mainly as a diagnostic method to monitor the neuronal pathway development and reorganization associated with injury (reviewed by Frye *et al.*^52^). Recent studies have also shown long-term positive outcomes of TMS therapy in autonomic dysfunctions and improvement in the characteristic behaviors associated with autism spectrum disorder in children^53^.

Nevertheless, the most serious risk associated with TMS treatment in TBI patients is induction of seizures. However, based on previous studies of TMS safety in adults and children, this appears to be a rare complication^54^,^55^,^56^,^57^,^58^,^59^. In line with this literature, in our study, during the animals’ daily monitoring, imaging, behavioral testing and TMS therapy sessions, we did not observe any seizures in any of the TBI animals subjected to TMS therapy. A thorough investigation of possible complications and a determination of safety guidelines for TMS therapy in TBI in pediatric and adult animal models of TBI, could facilitate the translation of this technique into the clinical setting.

Capitalizing on the preliminary success of TMS in alleviating comorbidities in TBI patients, and its apparent safety in children, our results demonstrate that TMS therapy in pediatric TBI rats was successful in improving long-term cortical neuronal functions to levels approximating those of healthy age-matched controls. The exact mechanism by which TMS affects the long-term neuronal firing rate remains to be determined. Concurrent electrophysiology recordings with TMS in non-human primates^60^ and in brain slices^61^ have shown that TMS induced immediate increases in cortical neuronal firing rates. While the exact mechanism by which TMS modifies long-term excitability remains largely unknown, it emerges that TMS can affect neuronal firing rates via several mechanisms. For example, studies in humans showed that high-frequency TMS increased cortical excitability in a way reminiscent of LTP^62^, and studies in rodents demonstrated that high-frequency TMS affected the long-term cortical activity of inhibitory interneurons via changes in the expression of calcium binding proteins, which, in turn, could modify network excitability levels^63^. It has also been shown that repetitive magnetic stimulation induces a long-lasting increase in glutamatergic synaptic strength in brain slices, suggesting the structural plasticity of excitatory synapses^64^.

Our results here and in previous studies^11^ demonstrate that CCI in young rats results in significant decreases in excitatory neuronal activity across the depth of the non-injured cortex. While the basis of the neuronal hypoactivity is not clear, both the increase in inhibition and the decrease in excitatory neurotransmission can impact LTP and long-term depression, have been associated with impaired plasticity described in humans as diaschisis^65^ as well as impaired synaptogenesis in the developing brain^20^. It is conceivable that the multiple TMS sessions in the TBI rats induced long-lasting increases in excitability in those neurons located in the non-injured cortex, which compensated for or overcame the neuronal hypoactivity. This has been manifested by increases in the expression of neuroplasticity markers, and increases in the firing rate and synaptic activity, measured by MUA and LTP, respectively. In addition, TMS therapy restored event-related fMRI responses in S1, demonstrating that fMRI is sensitive enough to monitor changes in the excitation levels associated with injury and subsequent therapy. Furthermore, TBI rats exhibited behavioral hyperactivity that has been shown to be associated with cortical injuries in animal models^30^,^46^,^47^, and is a common complication in pediatric TBI patients^48^,^49^,^50^,^66^,^67^. This hyperactivity was significantly reduced in TBI rats subjected to TMS therapy compared to TBI rats that did not receive TMS therapy. Noteworthy, a previous study demonstrated that high-frequency TMS has a greater potential to induce the expression of neuroplasticity markers when performed in awake rodents, compared to anesthetized rodents^68^. Thus, it is conceivable that high-frequency TMS protocol employed in the current study might have a greater effect in terms of behavioral outcome when translated to awake animals and patients.

In conclusion, our results suggest that TMS can guide plasticity and facilitate rehabilitation after pediatric TBI. Thisintervention could be readily translated to the clinical setting. While the developing brain has more potential for potentiating plasticity mechanisms, and thus, may be sensitive to TMS modulation, it is likely that TMS will have a positive outcome on neurorehabilitation in the adult TBI population as well.

## Methods

All animal procedures were conducted in accord with the National Institutes of Health (NIH) Guide for the Care and Use of Laboratory Animals and were approved by the Johns Hopkins University Animal Care and Use Committee.

### Animal model

TBI was induced using a CCI device (Pittsburgh Precision Instruments, Inc., Pennsylvania, USA) and was performed on 20 male Sprague-Dawley rats at postnatal day 16 (30–45 g)^32^. During the surgical procedure, anesthesia was maintained with 2% isoflurane. The rat’s head was fixed in a stereotaxic frame, and a rectal probe was then placed. After a left parietal craniotomy, and a 20-minute period of temperature stabilization, CCI was induced in the exposed cortex using a 6-mm flat metal impactor tip (speed, 5.5 m/s; duration, 50 ms; depth, 1.5 mm). The craniotomy was then resealed with an acrylic mixture, and the scalp incision was closed with interrupted sutures. An additional age-matched group of six rats served as controls.

### TMS treatment

TMS was applied using either a standard figure eight human coil (*n* = *5*; double 70 mm remote control coil, Magstim, Whitland, UK) or a figure eight rodent coil (*n* = *5*; 25 mm small double coil, Magstim) at 25% of the maximal output of a compact magnetic stimulator unit (Rapid2, Magstim). Different coils were applied in order to test the potential outcomes of TMS in both laboratorial and clinical settings, which assist its translation into a successful treatment. Since the effects of different coils was similar on means and standard deviations of results we combined the human coil and rodent coil results into a single TBI + TMS group for analysis. During stimulation, rats were anesthetized with 2% isoflurane. The coil was placed ~5 mm above the right, non-injured S1. The stimulation consisted of 9 trains of 100 pulses delivered at a frequency of 20 Hz and an inter-train rest time of 55 s to allow effective cooling of the coil. The entire stimulation protocol required nine minutes. It was previously shown that this TMS protocol induces long-lasting increases in markers for neuroplasticity^68^. The protocol was applied in TBI rats (*n* = *10*; TBI + TMS group) twice a week, starting nine days after the TBI procedure, for four weeks. Of note, similar or perhaps even more aggressive high frequency TMS protocols have been deemed safe and approved by the FDA for the treatment of depression^69^. The control groups consisted of TBI rats (*n* = *10*; TBI group) and healthy rats (*n* = *6*; control group) that were anesthetized and secured in the stereotaxic frame, but TMS pulses were not delivered. All animals were randomly allocated into groups. The number of rats designated for the experiments was based on our experience with the variability of animal physiology, and the requirement to reach statistical significance based on a power analysis using the hypothesis test of non-inferiority on group means for the average amplitude of evoked electrophysiological and fMRI responses.

### Extracellular electrophysiological recordings and data analysis

Rats were anesthetized using urethane (1.25 g/kg body weight, i.p.), then fixed in a stereotaxic frame. A 1 mm^2^ craniotomy window centered at AP, 0.0 mm and ML, 3.6 mm was made over the non-injured right S1. Two needle electrodes were inserted into the left forepaw to deliver electrical stimulation. Electrical stimulation was applied in three trains of 60 pulses (3 Hz, 0.3 mA, and 0.3 ms) with an inter-train rest time of 90 s. An axial array microelectrode (FHC, Inc.) was inserted through the center of the craniotomy window into S1 until reaching the depth of 1800 μm below the pia mater. Along the shank of the microelectrode, 12 sites spaced at 150 μm covered the whole depth of the rat cortex (starting at 150 μm). MUA and LFP were collected with a Cambridge Electronic Design interface (Micro1401-3, CED, Cambridge, UK) and Spike2 software (version 7, CED) with amplifiers (HiZx8, FHC, Inc.), and were sampled at 11 and 1 KHz, respectively. MUA signal was then band-pass filtered between 500 and 5000 Hz. Spiking activity was defined when the amplitude of the signal was greater than four times the standard deviation, which was calculated for 500 ms MUA before electrical stimulation. In order to evaluate stimulus-evoked spiking activity, a post-stimulus time histogram analysis was performed by event correlation analysis of the spiking with the tactile stimulation using 5 ms bins. MUAs were summed for each layer in every individual rat and averaged across the group. LFP waveforms were band-pass filtered between 0.1 and 300 Hz, and averaged with respect to the tactile stimulation and the mean amplitude of the negative deflection was calculated for each rat and averaged across the group.

### fMRI acquisition and data analysis

During fMRI measurements, rats were lightly anesthetized with dexmedetomidine (0.1 mg/kg/h, SC) which was shown to preserve neurovascular coupling^70^,^71^. Respiration rate, heart rate, rectal temperature, and partial pressure of oxygen were continuously monitored throughout all measurements (Starr Life Sciences, Pennsylvania, USA). Blood-oxygenation-level-dependent (BOLD) fMRI responses to contralateral tactile limb stimulation were measured in an ultra-high field of an 11.7 Tesla/16 cm horizontal bore small-animal scanner (Bruker BioSpin, Rheinstetten, Germany). A 72-mm quadrature volume coil and a 15-mm-diameter surface coil were used to transmit and receive magnetic resonance signals, respectively. For BOLD fMRI, gradient echo, echo planar imaging was used with a resolution of 150 × 150 × 1000 μm. Five 1 mm thick coronal slices covering S1 were acquired (effective echo time (TE), 11 ms; repetition time (TR), 1000 ms; bandwidth, 250 KHz; field of view (FOV), 1.92 × 1.92 cm; matrix size, 128 × 128). A T2-weighted RARE sequence was used to acquire high-resolution anatomical images (TE, 10 ms; TR, 5000 ms; bandwidth, 250 KHz; FOV, 1.92 × 1.92 cm; matrix size, 256 × 256) corresponding to the BOLD fMRI measurements. An fMRI block design was used, and stimulation of the left forepaw was conducted in a manner similar to that described above. The FMRIB Software Library (FSL, version 4.1.9) was used for analysis and spatial normalization^72^. Activation maps were obtained using the general linear model. Z-score statistics were cluster-size thresholded for an effective significance of *P* < 0.05. The activation threshold was set at 2.3.

### Open field test

Behavioral tests were performed by two independent examiners who were blind to the experimental groups. The open field arena was 24 inches (length) × 14 inches (height) × 18.5 inches (width). Each rat was placed in the center of the open field, and the test session lasted 10 minutes. During the session, the open field was isolated from the observer, and the light intensity in the open field arena was maintained stable. The rat was monitored by a camera (HDC-HS250, Panasonic, Osaka, Japan) during the test. Total distance and averaged velocity of the animals’ locomotor activities, and the time spent in the periphery were recorded and analyzed by an automated tracking system (CleverSys Inc., Virginia, USA).

### Forelimb and hindlimb reflex test

To test the forelimb strength and reflexes, the rat was lifted by the tail. The hindlimb test was initiated by placing the animal on a flat surface and pulling it upward gently by its tail while the forelimbs remain on the surface. The extension of the limb and the toes were observed as well as signs of abnormal movement and spasm^73^.

### Immunostaining

Immunostaining was performed on the brain slices of animal that were stimulated using the rodent coil. Rats were sacrificed and perfused with 4% PFA. Thirty-micron sections that were near bregma 0 were selected and washed three times, for five minutes each in PBS. Blocking was performed using 10% normal goat serum (Sigma, Missouri, USA) in PBS for one hour. The sections were incubated with the primary antibody anti-CaMKII, 1:200 (Millipore, Massachusetts, USA) in antibody diluent solution (Invitrogen, New York, USA) overnight. Sections were washed three times, for 15 minutes each with PBS, and incubated with the secondary antibody Alexa 488 anti-rat, 1:200 (Life Technologies, New York, USA) for three hours. Images were acquired using a Zeiss microscope (Axio Imager, Carl Zeiss, Inc., New York, USA) and then quantitatively analyzed with ImageJ software (NIH).

### Statistical analysis

All samples were included in the analysis. One-way analysis of variance (ANOVA) was applied to test the null hypothesis that the means of several populations were all equal. A post-hoc analysis was performed with a Scheffe *F* test to test differences across groups in the average amplitude of evoked neurophysiological responses for each layer, number of pixels in the fMRI activation map, the fluorescence intensity and the results from the open field tests. Significance was set at *P* < 0.05. All data were expressed as mean ± SEM.

## Additional Information

**How to cite this article**: Lu, H. *et al.* Transcranial magnetic stimulation facilitates neurorehabilitation after pediatric traumatic brain injury. *Sci. Rep.* **5**, 14769; doi: 10.1038/srep14769 (2015).

## Figures and Tables

**Table 1:** Quantification of the DAPI-stained and CaMKII-stained cells per mm^2^ in the control (*n* = *2*), TBI (*n* = *2*) and TBI + TMS (*n* = *2*) rats across different layers of non-injured S1.

	**L2–3**		**L4**	
	**DAPI**	**CamKII**	**DAPI**	**CaMKII**
Control	2125	1425	2198	1421
TBI	1949	1183	2096	1361
TBI + TMS	1926	1382	2068	1428

Data was averaged from two brain slices for each group with a field of view of 0.6 mm^2^.

## References

[b1] Xiong Y., Mahmood A. & Chopp M. Animal models of traumatic brain injury. Nat Rev Neurosci 14, 128–142 (2013). 23329160 10.1038/nrn3407 PMC3951995

[b2] Walker P. A. *et al.* Modern approaches to pediatric brain injury therapy. J Trauma 67, S120–127 (2009). 19667844 10.1097/TA.0b013e3181ad323a PMC2874892

[b3] Popernack M. L., Gray N. & Reuter-Rice K. Moderate-to-Severe Traumatic Brain Injury in Children: Complications and Rehabilitation Strategies. J Pediatr Health Care 29, e1–e7 (2014). 25449002 10.1016/j.pedhc.2014.09.003 PMC4409446

[b4] Cantore L., Norwood K. & Patrick P. Medical aspects of pediatric rehabilitation after moderate to severe traumatic brain injury. NeuroRehabilitation 30, 225–234 (2012). 22635128 10.3233/NRE-2012-0749

[b5] Barlow K. M., Thomson E., Johnson D. & Minns R. A. Late neurologic and cognitive sequelae of inflicted traumatic brain injury in infancy. Pediatrics 116, e174–185 (2005). 16061571 10.1542/peds.2004-2739

[b6] Yeates K. O. *et al.* Short- and long-term social outcomes following pediatric traumatic brain injury. J Int Neuropsychol Soc 10, 412–426 (2004). 15147599 10.1017/S1355617704103093

[b7] Wechsler B., Kim H., Gallagher P. R., DiScala C. & Stineman M. G. Functional status after childhood traumatic brain injury. J Trauma 58, 940–949 (2005). 15920407 10.1097/01.ta.0000162630.78386.98

[b8] Rosema S. *et al.* The trajectory of long-term psychosocial development 16 years following childhood traumatic brain injury. J Neurotrauma 32, 976–983 (2015). 25590265 10.1089/neu.2014.3567

[b9] Wilde E. A. *et al.* Diffusion tensor imaging in the corpus callosum in children after moderate to severe traumatic brain injury. J Neurotrauma 23, 1412–1426 (2006). 17020479 10.1089/neu.2006.23.1412

[b10] Ewing-Cobbs L. *et al.* Arrested development and disrupted callosal microstructure following pediatric traumatic brain injury: relation to neurobehavioral outcomes. Neuroimage 42, 1305–1315 (2008). 18655838 10.1016/j.neuroimage.2008.06.031 PMC2615227

[b11] Li N. *et al.* Evidence for impaired plasticity after traumatic brain injury in the developing brain. J Neurotrauma 31, 395–403 (2014). 24050267 10.1089/neu.2013.3059 PMC3922417

[b12] Arango J. I. *et al.* Posttraumatic seizures in children with severe traumatic brain injury. Childs Nerv Syst 28, 1925–1929 (2012). 22843174 10.1007/s00381-012-1863-0

[b13] Matsumoto J. H. *et al.* Prevalence of epileptic and nonepileptic events after pediatric traumatic brain injury. Epilepsy Behav 27, 233–237 (2013). 23480860 10.1016/j.yebeh.2013.01.024

[b14] Statler K. D. *et al.* A potential model of pediatric posttraumatic epilepsy. Epilepsy Res 86, 221–223 (2009). 19520549 10.1016/j.eplepsyres.2009.05.006 PMC2702176

[b15] Ip E. Y., Giza C. C., Griesbach G. S. & Hovda D. A. Effects of enriched environment and fluid percussion injury on dendritic arborization within the cerebral cortex of the developing rat. J Neurotrauma 19, 573–585 (2002). 12042093 10.1089/089771502753754055

[b16] D’Ambrosio R., Maris D. O., Grady M. S., Winn H. R. & Janigro D. Selective loss of hippocampal long-term potentiation, but not depression, following fluid percussion injury. Brain Res 786, 64–79 (1998). 9554957 10.1016/s0006-8993(97)01412-1

[b17] Prince D. A. & Tseng G. F. Epileptogenesis in chronically injured cortex: *in vitro* studies. J Neurophysiol 69, 1276–1291 (1993). 8492163 10.1152/jn.1993.69.4.1276

[b18] Ping X. & Jin X. Transition from initial hypoactivity to hyperactivity in cortical layer V pyramidal neurons following traumatic brain injury *in vivo*. J Neurotrauma (2015). 10.1089/neu.2015.3913 PMC4761811 26095991

[b19] Johnstone V. P., Shultz S. R., Yan E. B., O’Brien T. J. & Rajan R. The acute phase of mild traumatic brain injury is characterized by a distance-dependent neuronal hypoactivity. J Neurotrauma 31, 1881–1895 (2014). 24927383 10.1089/neu.2014.3343 PMC4224042

[b20] Johnston M. V. Plasticity in the developing brain: implications for rehabilitation. Dev Disabil Res Rev 15, 94–101 (2009). 19489084 10.1002/ddrr.64

[b21] Schulz R., Gerloff C. & Hummel F. C. Non-invasive brain stimulation in neurological diseases. Neuropharmacology 64, 579–587 (2013). 22687520 10.1016/j.neuropharm.2012.05.016

[b22] Celnik P. A. & Cohen L. G. Modulation of motor function and cortical plasticity in health and disease. Restor Neurol Neurosci 22, 261–268 (2004). 15502270

[b23] Lefaucheur J. P. *et al.* Neurogenic pain relief by repetitive transcranial magnetic cortical stimulation depends on the origin and the site of pain. J Neurol Neurosurg Psychiatry 75, 612–616 (2004). 15026508 10.1136/jnnp.2003.022236 PMC1739005

[b24] Nardone R. *et al.* Invasive and non-invasive brain stimulation for treatment of neuropathic pain in patients with spinal cord injury: a review. J Spinal Cord Med 37, 19–31 (2013). 24090372 10.1179/2045772313Y.0000000140 PMC4066547

[b25] Leo R. J. & Latif T. Repetitive transcranial magnetic stimulation (rTMS) in experimentally induced and chronic neuropathic pain: a review. J Pain 8, 453–459 (2007). 17434804 10.1016/j.jpain.2007.01.009

[b26] Demirtas-Tatlidede A., Vahabzadeh-Hagh A. M., Bernabeu M., Tormos J. M. & Pascual-Leone A. Noninvasive brain stimulation in traumatic brain injury. J Head Trauma Rehabil 27, 274–292 (2012). 21691215 10.1097/HTR.0b013e318217df55 PMC3342413

[b27] Villamar M. F., Santos Portilla A., Fregni F. & Zafonte R. Noninvasive brain stimulation to modulate neuroplasticity in traumatic brain injury. Neuromodulation 15, 326–338 (2012). 22882244 10.1111/j.1525-1403.2012.00474.x

[b28] Pape T. L., Rosenow J. & Lewis G. Transcranial magnetic stimulation: a possible treatment for TBI. J Head Trauma Rehabil 21, 437–451 (2006). 16983227 10.1097/00001199-200609000-00063

[b29] Adelson P. D., Whalen M. J., Kochanek P. M., Robichaud P. & Carlos T. M. Blood brain barrier permeability and acute inflammation in two models of traumatic brain injury in the immature rat: a preliminary report. Acta Neurochir Suppl 71, 104–106 (1998). 9779157 10.1007/978-3-7091-6475-4_31

[b30] Pullela R. *et al.* Traumatic injury to the immature brain results in progressive neuronal loss, hyperactivity and delayed cognitive impairments. Dev Neurosci 28, 396–409 (2006). 16943663 10.1159/000094166

[b31] Scafidi S., Racz J., Hazelton J., McKenna M. C. & Fiskum G. Neuroprotection by acetyl-L-carnitine after traumatic injury to the immature rat brain. Dev Neurosci 32, 480–487 (2010). 21228558 10.1159/000323178 PMC3073762

[b32] Robertson C. L., Saraswati M. & Fiskum G. Mitochondrial dysfunction early after traumatic brain injury in immature rats. J Neurochem 101, 1248–1257 (2007). 17403141 10.1111/j.1471-4159.2007.04489.x

[b33] Prins M. L., Povlishock J. T. & Phillips L. L. The effects of combined fluid percussion traumatic brain injury and unilateral entorhinal deafferentation on the juvenile rat brain. Brain Res Dev Brain Res 140, 93–104 (2003). 12524180 10.1016/s0165-3806(02)00588-6

[b34] Siebner H. R. & Rothwell J. Transcranial magnetic stimulation: new insights into representational cortical plasticity. Exp Brain Res 148, 1–16 (2003). 12478392 10.1007/s00221-002-1234-2

[b35] Cowen T. D. *et al.* Influence of early variables in traumatic brain injury on functional independence measure scores and rehabilitation length of stay and charges. Arch Phys Med Rehabil 76, 797–803 (1995). 7668948 10.1016/s0003-9993(95)80542-7

[b36] Adelson P. D., Dixon C. E. & Kochanek P. M. Long-term dysfunction following diffuse traumatic brain injury in the immature rat. J Neurotrauma 17, 273–282 (2000). 10776912 10.1089/neu.2000.17.273

[b37] Scheibel R. S. *et al.* Altered brain activation during cognitive control in patients with moderate to severe traumatic brain injury. Neurorehabil Neural Repair 21, 36–45 (2007). 17172552 10.1177/1545968306294730

[b38] Christodoulou C. *et al.* Functional magnetic resonance imaging of working memory impairment after traumatic brain injury. J Neurol Neurosurg Psychiatry 71, 161–168 (2001). 11459886 10.1136/jnnp.71.2.161 PMC1737512

[b39] Chen J. K. *et al.* Functional abnormalities in symptomatic concussed athletes: an fMRI study. Neuroimage 22, 68–82 (2004). 15109998 10.1016/j.neuroimage.2003.12.032

[b40] Lovell M. R. *et al.* Functional brain abnormalities are related to clinical recovery and time to return-to-play in athletes. Neurosurgery 61, 352–359 (2007). 17762748 10.1227/01.NEU.0000279985.94168.7F

[b41] Newsome M. R. *et al.* Brain activation during working memory after traumatic brain injury in children. Neurocase 13, 16–24 (2007). 17454685 10.1080/13554790601186629

[b42] Niskanen J. P. *et al.* Monitoring functional impairment and recovery after traumatic brain injury in rats by FMRI. J Neurotrauma 30, 546–556 (2013). 23259713 10.1089/neu.2012.2416 PMC3636591

[b43] Mishra A. M. *et al.* Decreased resting functional connectivity after traumatic brain injury in the rat. PLoS One 9, e95280 (2014). 24748279 10.1371/journal.pone.0095280 PMC3991600

[b44] Max J. E. *et al.* Anxiety disorders in children and adolescents in the first six months after traumatic brain injury. J Neuropsychiatry Clin Neurosci 23, 29–39 (2011). 21304136 10.1176/jnp.23.1.jnp29

[b45] Li L. & Liu J. The effect of pediatric traumatic brain injury on behavioral outcomes: a systematic review. Dev Med Child Neurol 55, 37–45 (2013). 22998525 10.1111/j.1469-8749.2012.04414.x PMC3593091

[b46] Ajao D. O. *et al.* Traumatic brain injury in young rats leads to progressive behavioral deficits coincident with altered tissue properties in adulthood. J Neurotrauma 29, 2060–2074 (2012). 22697253 10.1089/neu.2011.1883 PMC3408248

[b47] Semple B. D., Canchola S. A. & Noble-Haeusslein L. J. Deficits in social behavior emerge during development after pediatric traumatic brain injury in mice. J Neurotrauma 29, 2672–2683 (2012). 22888909 10.1089/neu.2012.2595 PMC3510450

[b48] Bloom D. R. *et al.* Lifetime and novel psychiatric disorders after pediatric traumatic brain injury. J Am Acad Child Adolesc Psychiatry 40, 572–579 (2001). 11349702 10.1097/00004583-200105000-00017

[b49] Konrad K., Gauggel S., Manz A. & Scholl M. Inhibitory control in children with traumatic brain injury (TBI) and children with attention deficit/hyperactivity disorder (ADHD). Brain Inj 14, 859–875 (2000). 11076133 10.1080/026990500445691

[b50] Giza C. C. & Prins M. L. Is being plastic fantastic? Mechanisms of altered plasticity after developmental traumatic brain injury. Dev Neurosci 28, 364–379 (2006). 16943660 10.1159/000094163 PMC4297630

[b51] Koski L. *et al.* Noninvasive brain stimulation for persistent postconcussion symptoms in mild traumatic brain injury. J Neurotrauma 32, 38–44 (2015). 24955920 10.1089/neu.2014.3449

[b52] Frye R. E., Rotenberg A., Ousley M. & Pascual-Leone A. Transcranial magnetic stimulation in child neurology: current and future directions. J Child Neurol 23, 79–96 (2008). 18056688 10.1177/0883073807307972 PMC2539109

[b53] Casanova M. F. *et al.* Effects of weekly low-frequency rTMS on autonomic measures in children with autism spectrum disorder. Front Hum Neurosci 8, 851 (2014). 25374530 10.3389/fnhum.2014.00851 PMC4204613

[b54] Quintana H. Transcranial magnetic stimulation in persons younger than the age of 18. J ECT 21, 88–95 (2005). 15905749 10.1097/01.yct.0000162556.02720.58

[b55] Wassermann E. M. *et al.* Use and safety of a new repetitive transcranial magnetic stimulator. Electroencephalogr Clin Neurophysiol 101, 412–417 (1996). 8913194

[b56] Chen R. *et al.* Safety of different inter-train intervals for repetitive transcranial magnetic stimulation and recommendations for safe ranges of stimulation parameters. Electroencephalogr Clin Neurophysiol 105, 415–421 (1997). 9448642 10.1016/s0924-980x(97)00036-2

[b57] Pascual-Leone A. *et al.* Safety of rapid-rate transcranial magnetic stimulation in normal volunteers. Electroencephalogr Clin Neurophysiol 89, 120–130 (1993). 7683602 10.1016/0168-5597(93)90094-6

[b58] Garvey M. A. & Gilbert D. L. Transcranial magnetic stimulation in children. Eur J Paediatr Neurol 8, 7–19 (2004). 15023371 10.1016/j.ejpn.2003.11.002

[b59] Rossi S., Hallett M., Rossini P. M. & Pascual-Leone A. Safety, ethical considerations, and application guidelines for the use of transcranial magnetic stimulation in clinical practice and research. Clin Neurophysiol 120, 2008–2039 (2009). 19833552 10.1016/j.clinph.2009.08.016 PMC3260536

[b60] Mueller J. K. *et al.* Simultaneous transcranial magnetic stimulation and single-neuron recording in alert non-human primates. Nat Neurosci 17, 1130–1136 (2014). 24974797 10.1038/nn.3751 PMC4115015

[b61] Pashut T. *et al.* Patch-clamp recordings of rat neurons from acute brain slices of the somatosensory cortex during magnetic stimulation. Front Cell Neurosci 8, 145 (2014). 24917788 10.3389/fncel.2014.00145 PMC4042461

[b62] Pascual-Leone A., Valls-Sole J., Wassermann E. M. & Hallett M. Responses to rapid-rate transcranial magnetic stimulation of the human motor cortex. Brain 117 (Pt 4), 847–858 (1994). 7922470 10.1093/brain/117.4.847

[b63] Benali A. *et al.* Theta-burst transcranial magnetic stimulation alters cortical inhibition. J Neurosci 31, 1193–1203 (2011). 21273404 10.1523/JNEUROSCI.1379-10.2011 PMC6623597

[b64] Vlachos A. *et al.* Repetitive magnetic stimulation induces functional and structural plasticity of excitatory postsynapses in mouse organotypic hippocampal slice cultures. J Neurosci 32, 17514–17523 (2012). 23197741 10.1523/JNEUROSCI.0409-12.2012 PMC6621866

[b65] Duffau H. Brain plasticity: from pathophysiological mechanisms to therapeutic applications. J Clin Neurosci 13, 885–897 (2006). 17049865 10.1016/j.jocn.2005.11.045

[b66] Di Battista A., Godfrey C., Soo C., Catroppa C. & Anderson V. Depression and health related quality of life in adolescent survivors of a traumatic brain injury: a pilot study. PLoS One 9, e101842 (2014). 25010719 10.1371/journal.pone.0101842 PMC4092017

[b67] Karver C. L. *et al.* Age at injury and long-term behavior problems after traumatic brain injury in young children. Rehabil Psychol 57, 256–265 (2012). 22946613 10.1037/a0029522 PMC3750969

[b68] Gersner R., Kravetz E., Feil J., Pell G. & Zangen A. Long-term effects of repetitive transcranial magnetic stimulation on markers for neuroplasticity: differential outcomes in anesthetized and awake animals. J Neurosci 31, 7521–7526 (2011). 21593336 10.1523/JNEUROSCI.6751-10.2011 PMC6622610

[b69] Yukimasa T. *et al.* High-frequency repetitive transcranial magnetic stimulation improves refractory depression by influencing catecholamine and brain-derived neurotrophic factors. Pharmacopsychiatry 39, 52–59 (2006). 16555165 10.1055/s-2006-931542

[b70] Li N., van Zijl P., Thakor N. & Pelled G. Study of the Spatial Correlation Between Neuronal Activity and BOLD fMRI Responses Evoked by Sensory and Channelrhodopsin-2 Stimulation in the Rat Somatosensory Cortex. J Mol Neurosci 53, 553–561 (2014). 24443233 10.1007/s12031-013-0221-3 PMC4104155

[b71] Pawela C. P. *et al.* Resting-state functional connectivity of the rat brain. Magn Reson Med 59, 1021–1029 (2008). 18429028 10.1002/mrm.21524 PMC2562321

[b72] Smith S. M. *et al.* Advances in functional and structural MR image analysis and implementation as FSL. Neuroimage 23 Suppl 1, S208–219 (2004). 15501092 10.1016/j.neuroimage.2004.07.051

[b73] Fujimoto S. T. *et al.* Motor and cognitive function evaluation following experimental traumatic brain injury. Neurosci Biobehav Rev 28, 365–378 (2004). 15341032 10.1016/j.neubiorev.2004.06.002

